# Use of Continuous Glucose Monitoring in Non-ICU Hospital Settings for People With Diabetes: A Scoping Review of Emerging Benefits and Issues

**DOI:** 10.1177/19322968211053652

**Published:** 2021-10-25

**Authors:** Benjamin Clubbs Coldron, Vivien Coates, Amjed Khamis, Sandra MacRury

**Affiliations:** 1 Division of Rural Health and Wellbeing, Centre for Health Science, University of Highlands and Islands, Inverness, UK; 2 School of Nursing, Ulster University, Derry, UK; 3 Western Health and Social Care Trust, Altnagelvin Area Hospital, Londonderry, UK; 4 Letterkenny University Hospital, Letterkenny, Ireland; 5 Raigmore Hospital, Inverness, UK

**Keywords:** diabetes mellitus, continuous glucose monitoring, inpatient care, flash glucose monitoring

## Abstract

**Background:**

Evidence indicates that poor glycemic control is associated with increased morbidity and length of stay in hospital. There are a wide range of guidelines published, which seek to ensure safe and effective inpatient glycemic control in the hospital setting. However, the implementation of these protocols is limited in practice. In particular, the feasibility of “flash” and continuous glucose monitoring (CGM) remains untested on general wards.

**Method:**

Scoping Review.

**Results:**

If used in the general ward hospital settings, CGM and flash glucose monitoring (FGM) systems could lead to improved glycemic control, decreased length of stay, and reduced risk of severe hypoglycemia or hyperglycemia. Potential problems include lack of experience with this technology and costs of sensors. Rapid analysis of glucose measurements can facilitate clinical decision making and therapy adjustment in the hospital setting. In addition, people with diabetes may be empowered to better self-manage their condition in hospital as they have direct access to their glucose data.

**Conclusions:**

More studies are required in which the feasibility, benefits and limitations of FGM and CGM in non–intensive care unit hospital settings are elucidated. We need evidence on which types of hospital wards might benefit from the introduction of this technology and the contexts in which they are less useful. We also need to identify the types of people who are most likely to find FGM and CGM useful for self-management and for which populations they have the most benefit in terms of clinical outcomes and length of stay.

## Introduction

The growing prevalence of diabetes across the world and the demand for elective and unscheduled hospital admissions resulting from diabetes complications mean a high percentage of hospital inpatients require sustained glucose monitoring.^1,2^ Poor glycemic control in hospital is associated with adverse clinical outcomes and increased length of stay.^3,4^ A high incidence of inpatient hyperglycemia and hypoglycemia represents a significant financial and practical burden for service users, health care providers, and their families and carers.^5^

Diabetes technology has progressed greatly over the past decade.^6^ Advances in glucose monitoring and insulin delivery systems have improved clinical outcomes and quality of life for people with type 1 diabetes (T1D) in the outpatient setting. Use of factory-calibrated subcutaneous glucose monitoring licensed for nonadjuvant insulin dosing has been shown to attenuate hypoglycemic risk while reducing the burden of capillary blood glucose testing.^7,8^ Automated insulin delivery systems, known as artificial pancreas or closed-loop, have been shown in free-living unsupervised home studies to improve glycemic control and to reduce the burden of hypoglycemia in people with T1D.^9,10^ Emerging data suggest that automated insulin delivery technology may also benefit inpatient hyperglycemia management in those with type 2 diabetes (T2D).^11,12^

In addition to traditional monitoring of blood glucose using point-of-care capillary blood glucose testing (POCT), there are now a wide variety of technologies available that allow continuous measurement of glucose levels in interstitial fluid.^1^ Devices that do this are called continuous glucose monitoring (CGM) or flash glucose monitoring (FGM). Devices on the market include FreeStyle Libre, Dexcom G4 Platinum, Dexcom G5 Mobile, Dexcom G6, Senseonics-Eversense, and Medtronic Guardian Connect. The key difference between CGM and FGM is that the former measures glucose levels continuously and sends data automatically to a monitor, smart device, or insulin pump.^2^ With CGM, service users and providers can set alerts for high or low glucose levels, or the rate of change. Continuous glucose monitoring devices often have to be calibrated twice daily. By contrast, FGM devices such as FreeStyle Libre and Dexcom G6 are factory-calibrated. In addition, they only provide an immediate reading when the sensor is scanned by a handset or smart phone with the relevant software app and show trends in the data. This must be done at least every 8 hours.^3^ FGM represents a potentially cost-effective solution, allowing near real-time, accurate, and accessible measurement of glucose levels without the need for frequent calibration.^4,5^

While the use of FGM and CGM in the community setting is rising and has demonstrated benefits on glycemic control, the utility of these devices in the hospital setting, and particularly outside the intensive care unit (ICU), is less well understood.^5^ Recent reviews and commentaries suggest that the use of FGM and CGM systems in the non-ICU inpatient setting could improve overall glycemic control and decrease hypoglycemia.^5,6^ Several FGM and CGM systems have been evaluated in the hospital setting, and interest in adopting these technologies more widely has grown during the COVID-19 pandemic.^7^ These technologies may be useful in increasing the efficiency of hospital care and reducing length of stay for people with diabetes.^5^ This has become particularly pertinent in light of the fact that diabetes increases the risk of hospitalization and death associated with coronavirus infection.^8^

The aim of this article is to provide an overview of the current status and future outlook of FGM and CGM in non-ICU care settings, to identify and describe evidence of potential benefits of FGM in hospital settings for adults with T1D and T2D diabetes as well as limitations of the technology and its application. In addition, we outline key gaps in the emerging literature and fruitful lines of inquiry for future research.

## Method

A scoping review was conducted to determine the scope of the literature around use of FGM and CGM in the non-ICU inpatient setting and to provide a detailed overview of the ways that these technologies are used, who they are applied to, where and when, as well as the potential benefits and limitations.^13^

An electronic search was conducted in January 2021 on MEDLINE, PubMed, Google scholar, EMBASE, CINAHL, and The Cochrane Library to review the available literature. We searched the titles and abstracts of papers, and the time period covered was from January 2010 to September 2021. Keywords included “Flash Glucose Monitor,” “Flash Glucose Monitoring,” “Continuous Glucose Monitor,” “Continuous Glucose Monitoring,” “FGM,” “CGM,” “Inpatient,” and “Hospital.” Publications that examined CGM and FGM use in the non-ICU setting were included, and those in the ICU setting were excluded from this review article because there is abundant research in this context already. We also excluded “closed-loop” systems of glucose monitoring. A closed-loop system uses a smart phone app to automatically adjust the insulin delivery on an insulin pump based on glucose readings from a continuous glucose sensor.^5^ As these devices are not yet tried and tested technologies, are more sophisticated and expensive, and use in the inpatient setting has not been evaluated, we focused on continuous glucose measuring devices alone.^5^ Only papers reporting primary research were included; those reporting commentary and reviews were excluded. The target populations under study in the various articles covered T1D and T2D. We excluded articles that focused exclusively on children or the critically ill. Articles included in the review came from the United States, Europe, the United Kingdom, Australia, Austria, Japan, and Canada.

In analyzing the literature gathered, we used thematic analysis that allowed us to identify common benefits and issues across the literature and to outline how the introduction for CGM and FGM technologies affects the provision of care in non-ICU hospital settings.

## Results

**Figure 1.**
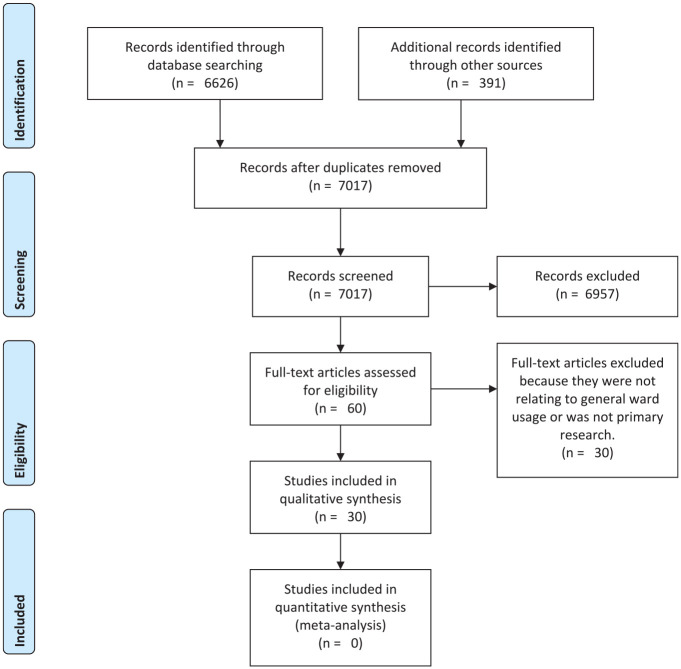
Use of flash glucose monitoring in non–intensive care unit hospital settings for people with diabetes: a scoping review of emerging benefits and issues—Preferred Reporting Items for Systematic Reviews and Meta-Analyses flow diagram.

We identified 30 primary research papers relevant to the topic. The process of selection is detailed in the Preferred Reporting Items for Systematic Reviews and Meta-Analyses (PRISMA) flow diagram (Figure 1). Most of the papers detailed observational studies, retrospective studies, or clinical trials (see Supplementary Table 1).

### Potential Benefits of FGM and CGM in Non-ICU Hospital Settings

#### Prevention

Existing evidence suggests that both FGM and CGM are accurate and reliable systems when applied to noncritical situations in hospital.^11,12^ Several clinical studies compared POCT with CGM in hospitalized patients and found various benefits of CGM use in the general ward settings.^14-20^ In one study in general wards, CGM detected 15 times more nocturnal hypoglycemic episodes (defined by the study team as <3.9 mmol/L) than those detected by POCT.^21^ The number of hyperglycemic episodes (defined by the study team as >13.9 mmol/L) detected by CGM was also 12.5 times greater than that by POCT. In observational studies, a higher number of hypoglycemic episodes were also detected by CGM compared with POCT.^21^ Although some studies showed no difference in mean daily glucose concentrations, significantly greater numbers of hypoglycemic and hyperglycemic events were detected. This highlights CGM as a potentially valuable tool in detecting and preventing hypoglycemic events at times when POCT is less frequently conducted, especially in inpatients known to be at increased risk of hypoglycemia.^22^ The increased detection of adverse glycemic events has been found in both T1D populations requiring insulin and T2D populations on basal-bolus and subcutaneous insulin in non-ICU settings.^21,22^

Use of CGM and FGM devices does not appear to increase the risk of adverse effects in hospital in relation to severe hypoglycemia or hyperglycemia.^18^ In outpatient settings, real-world data demonstrate that the use of CGM improves well-being and may be associated with a decreased disease burden.^21^ Although the costs and benefits require careful analysis, it appears that non-ICU usage of CGM and FGM could prove an effective preventive measure, which could ultimately make inpatient care for people with diabetes more efficient.^5^

#### Convenience

Further potential benefits of CGM and FGM include the fact that glucose levels can be monitored continuously throughout the day and night without the need for more painful and disruptive capillary blood glucose testing, potentially leading to improved patient satisfaction and added convenience for staff.^23,24^ Because patients and clinicians can monitor glucose levels at any time, trends in glucose levels can be observed (eg, relating to insulin administration and food intake) and corrective action can be taken earlier, behavior modified, and therapy, diet, and meal times tailored to specific persons’ needs.^5^ This also means that when a patient needs to be quarantined and isolated, blood glucose can be monitored remotely without the need for close contact.^25^

#### Glucose management

Two studies excluded from the review indicate that CGM and FGM can empower people with diabetes to better self-manage glucose levels outside the hospital setting.^26,27^ However we could find no data on whether this can be replicated in non-ICU inpatient settings where disempowerment can be an issue. A recent randomized controlled trial showed that glucose control by clinical staff may be enhanced.^28^ The trial, involving 110 adults with T2D, demonstrated that the CGM group had significantly lower mean glucose (M∆ = −18.5 mg/dL) and percentage of time in hyperglycemia >250 mg/dL (−11.41%) and higher time in the range of 70-250 mg/dL (+11.26%) compared with the control group (*P* < .05).^28^

Although a number of papers provide evidence that FGM and CGM usage in noncritical inpatient hospital wards can have benefits in improved glycemic control, it is not yet demonstrated that this can result in reduced length of stay.^5,28^ Continuous glucose monitoring devices and FreeStyle Libre 2 have an alarm that can be set to personalized high and low levels, thus increasing the chance that rapid change will be immediately detected. In addition, glucose telemetry systems allow remote monitoring and support more sophisticated management of glucose levels by clinical staff.^29^

Where the full potential of FGM and CGM data is realized, clinicians and service users may observe trends associated with food intake and insulin use so that preventive action can be taken and care can be tailored to specific needs.

As one of the main benefits of FGM and CGM usage is in tailoring and optimizing insulin therapy, the population of people with diabetes who are most frequently subject to these interventions are people with T1D.^5^ However, using this technology as a tool to improve glucose management in insulin-dependent people with T2D appears to be effective.^30^ There appear to be no studies yet undertaken investigating whether FGM and CGM could be used to individualize dietary advice and diabetes management plans for people with T1D and those with T2D who are diet controlled, but continuous blood glucose data could be useful for these purposes also.^31^

#### Implementation

Continuous glucose monitoring has been shown to be relatively simple to implement in general wards with the use of telemetry systems.^22,29^ Evidence suggests that FGM devices are user-friendly, easy to set up and insert, and generally well-reviewed by service users.^11^ The “on-demand” glucose data appear accessible for nurses and service users and provide real-time glucose monitoring which can be scanned at any time.

The FGM and CGM technologies facilitate more frequent and convenient monitoring of glucose levels in the non-ICU hospital setting. This means that FGM and CGM could be used to improve clinical decision making in non-ICU hospital settings potentially with much reduced use of POCT.^32^ This review provides evidence that, in the right settings and with engaged teams of health care professionals, this can lead to improved glycemic control and reduced incidences of hypoglycemia and hyperglycemia. These are a major contributor to prolonged hospital stay, so it follows logically that these devices offer the potential to improve inpatient care for people with diabetes and decrease length of stay.^5,11,28^

### Potential Issues of FGM and CGM Use in Non-ICU Hospital Settings

#### Technical issues

There is a time lag between interstitial fluid and blood glucose concentrations, and so rapidly changing glucose levels may give a misleading reading on some CGM devices.^33^ With devices such as the FreeStyle Libre, warning systems are in place to detect rapidly falling or increasing glucose to counter these issues, but in most jurisdictions capillary blood glucose tests are still preferred even if FGM or CGM devices are in use as this is the more accurate, reliable, and up-to-date measure.^6^ Blood glucose measures are also under the scrutiny of the hospital laboratories, so quality assurance processes are in place.

#### Cause and effect

At least one study has shown that without using CGM or FGM, early identification and management of inpatients with diabetes by specialist teams decreased hyperglycemia, and hospital-acquired infections increased.^31^ This indicates that it may not be the technology per se but the increased clinical attention that a patient attracts with greater glucose monitoring that could be a decisive factor. Further studies are needed to isolate the causal mechanism involved in improving outcomes for inpatients with diabetes and determine whether it is more cost-effective to invest in specialist staff rather than such devices.

#### Inexperience with the technology

Inexperience with the technology for both service users and clinicians may mean that the benefits of FGM and CGM use may be reduced. One recent study found that the development of protocols to use CGM trend arrows, alerts, and alarms is necessary to improve implementation.^33^ The use of glucose telemetry systems may be one way of improving and simplifying the process of surveillance; however, effectively interpreting and acting on this information will require training and staff engagement.^22,29^ The CGM devices will only have a positive impact if service users know how to read and act on out-of-range readings and if staff have appropriate training and protocols to follow. Glucose levels recorded from interstitial fluid often have to be verified by measuring blood glucose at present, so awareness of the time lag is vital. It is envisaged that, when appropriate, quality assurance processes are in place for CGM/FGM that POCT may no longer be required.

#### Expense

Additional limitations include costs related to supplies of FGM and CGM scanners and sensors. These can be expensive, and it is not clear whether increased efficiency, for example, reductions in length of stay, can offset the costs of the intervention.^5^ In addition to the costs of the devices, significant investment may be necessary for hospital staff training and for development of infrastructure to support inpatient use of CGM and FGM devices.^22,34^ The continuous measurement of glucose levels alongside POCT may create potential for an increased workload for nursing and clinical staff in the short term. In particular, diabetes specialist teams may find increased demand for advice and engagement based on the huge amount of data available to patients themselves and attendant nursing staff.^28^ The potential inaccuracy of interstitial glucose measurements due to medication interferences, sensor lag, or drift may mean that some of this increased demand may prove unnecessary and wasteful. Clinicians and service users may get overloaded with data, which might create unnecessary confusion or worry.

The FGM devices may not be appropriate for everyone and may be best targeted to those service users who need additional support to effectively self-manage their glucose.^35^ Some service users may experience skin irritation due to the adhesive or discomfort caused by wearing the device for prolonged periods in hospital.^36^ In addition, routine scans and procedures and medications applied in hospital mean that glucose sensors might have to be removed and replaced often to avoid interference.^5,17,28^

In noncritical care settings, both CGM and FGM have been associated with improved clinical outcomes, and there is a clear consensus in the clinical community that these devices have great potential.^5,6,11,37,38^ However, current evidence remains controversial and at times somewhat contradictory. A summary of potential benefits and limitations of FGM and CGM in hospital settings can be found in Table 1.

**Table 1.** Summary of Potential Benefits and Limitations of FGM and CGM in Non-ICU Hospital Settings.

Potential benefits of non-ICU use of CGM/FGMs	Potential limitations of non-ICU use of CGM/FGMs
May lead to improved glycemic control	Hospital stays tend to be short, and therefore any benefits may be short-lived
Decreased length of stay	Without more sustained usage after discharge, there may be a danger of readmission
Reduced risk of adverse events related to severe hypoglycemia or hyperglycemia	Sensor lag, or drift, may mean creating a false sense of security and resulting in avoidable adverse events
Glucose levels can be monitored 24 hours a day without disturbing the patient	Potential inaccuracy of interstitial glucose measurements due to medication or clinical procedures means that it may have to be removed and replaced periodically
Patients and clinicians can view glucose levels at times in between finger prick tests	May create increased workload for health care practitioners
Frequency of finger prick checks may be reduced	Some finger prick checks remain necessary as CGM and FGM measure interstitial fluid
Patients and clinicians can observe glucose trends, so action can be taken earlier	Inexperience with the technology may mean clinicians and service users get overloaded with data
Can empower inpatients to self-manage their glucose	Significant costs may be incurred for the sensors, for hospital staff/patient training, and for development of infrastructure to support inpatient use
Ease of application and use of FGM make it user-friendly and low-risk	Some experience skin irritation due to the adhesive on FGM or discomfort

Abbreviations: CGM, continuous glucose monitoring; FGM, flash glucose monitoring; ICU, intensive care unit.

#### Gaps in the literature

Questions remain as to whether FGM and CGM can be effective in reducing length of stay and whether it can improve clinical outcomes, patient self-management, and/or patient experiences. In addition, there is a lack of health economics perspectives and detailed cost-benefit analysis of non-ICU applications.

Often hospital inpatients have more significant care needs than people in the community and will therefore require additional assistance from staff to monitor and manage their glucose levels. More research is needed to determine what resources and policies are effective in meeting these needs and adapting FGM and CGM use to these contexts. Inpatients are also often dependent on staff to provide food, and flexibility around mealtimes is often reduced in hospital settings, partly due to infection prevention and control policies restricting food being brought in from outside the hospital. In addition, glucose sensors may be problematic for patients in hospital should they require medication/procedures that could interfere with glucose measurements. There are no sociological studies investigating the way the introduction of these novel technologies affects the ability of people with diabetes in hospital to self-manage their glucose levels and whether there are any beneficial or adverse effects on the power dynamics between service users and providers.

### Strengths and Limitations

In conducting this scoping review, there were several constraints and limitations. There is limited literature in the specific area of non-ICU hospital application of FGM and CGM. This meant that rather than a comprehensive synthesis of the evidence on FGM/CGM application in non-ICU hospital settings, we had to approach it as a scoping exercise that would enable us to review the available evidence and identify the gaps in the literature to inform future research. The quality of the studies included was not assessed, although all were peer-reviewed and published articles.

The picture is further complicated by the differences and similarities between CGM and FGM and “closed-loop” systems. The literature was difficult to assess in terms of comparisons in application to non-ICU settings as there is a broad overlap in the way these technologies are referred to in the literature. Conceptually speaking, “non-ICU hospital settings” cover a huge variety of very different contexts where the introduction of new technologies such as FGM and CGM might have widely divergent effects.

## Conclusion

Our findings point to the need for future research to plug gaps in the existing evidence base. More studies are required in which the feasibility, benefits, and limitations of FGM and CGM in non-ICU hospital settings are elucidated as well as evaluations of the impact on inpatient self-management. The current literature does not make it clear which types of hospital wards might benefit most from the introduction of this technology and the contexts in which the devices may be less useful. There is also a need to identify the characteristics of people who are most likely to benefit from FGM and CGM in terms of patient experiences, clinical outcomes, and length of stay. In addition, the health economics of FGM and CGM introduction needs to be comprehensively modeled so that we can understand whether the introduction of these novel devices is cost-effective and an efficient use of resources. Both FGM and CGM are tools that can facilitate clinical decision making and empower patients to self-manage their condition. However, without appropriate training, support, and institutional flexibility, their potential will never be realized.

## Supplemental Material

sj-docx-1-dst-10.1177_193229682111053652 – Supplemental material for Use of Continuous Glucose Monitoring in Non-ICU Hospital Settings for People With Diabetes: A Scoping Review of Emerging Benefits and Issues Click here for additional data file. Supplemental material, sj-docx-1-dst-10.1177_193229682111053652 for Use of Continuous Glucose Monitoring in Non-ICU Hospital Settings for People With Diabetes: A Scoping Review of Emerging Benefits and Issues by Benjamin Clubbs Coldron, Vivien Coates, Amjed Khamis and Sandra MacRury in Journal of Diabetes Science and Technology
