# Supplementary material for: Use of Continuous Glucose Monitoring in Non-ICU Hospital Settings for People With Diabetes: A Scoping Review of Emerging Benefits and Issues
Source: J Diabetes Sci Technol. 2021 Oct 25;17(2):467–73. doi: 10.1177/19322968211053652 (PMC10012381; doi:10.1177/19322968211053652)
Supplement: sj-docx-1-dst-10.1177_193229682111053652 – Supplemental material for Use of Continuous Glucose Monitoring in Non-ICU Hospital Settings for People With Diabetes: A Scoping Review of Emerging Benefits and Issues [file sj-docx-1-dst-10.1177_193229682111053652.docx]

**Supplementary Table 1: Summary of Scoping Review of Literature on** **FGM and CGM in non-ICU hospital settings**

| **Reference** | **Year** | **Jurisdiction** | **Notes** | **Setting** | **Target Population** |
| --- | --- | --- | --- | --- | --- |
| Dungan, KM, Han, W, Miele, A, Zeidan, T, Weiland, K. Determinants of the accuracy of continuous glucose monitoring in non-critically ill patients with heart failure or severe hyperglycemia. J Diabetes Sci Technol. 2012;6(4):884 | 2012 | USA | Hospitalized patients with congestive heart failure (CHF) exacerbation (receiving IV or subcutaneous insulin) or SH requiring insulin infusion were compared to outpatients referred for retrospective CGM. | Non-ICU general wards | T1D and T2D on subcutaneous insulin |
| Burt, MG, Roberts, GW, Aguilar-Loza, NR, Stranks, SN. Brief report: comparison of continuous glucose monitoring and finger-prick blood glucose levels in hospitalized patients administered basal-bolus insulin. Diabetes Technol Ther. 2013;15(3):241-245. | 2013 | Australia | Evaluated glycaemic control and accuracy. Finger prick testing underestimates the prevalence of postprandial hyperglycemia and hypoglycemia. | Non-ICU general wards | T1D and T2D on basal bolus insulin |
| Schaupp, L, Donsa, K, Neubauer, KM, et al. Taking a closer look—continuous glucose monitoring in non-critically Ill hospitalized patients with type 2 diabetes mellitus under basal-bolus insulin therapy. Diabetes Technol Ther. 2015;17(9):611-618. | 2015 | Austria | CGM and BG measurements were consistent, but there were a high number of missed hypo- and hyperglycemic episodes with BG measurements, particularly during nighttime. | Non-ICU general wards | T2D on Insulin |
| Ushigome E, Yamazaki M, Hamaguchi M, Ito T, Matsubara S, Tsuchido Y, Kasamatsu Y, Nakanishi M, Fujita N, Fukui M. Usefulness and Safety of Remote Continuous Glucose Monitoring for a Severe COVID-19 Patient with Diabetes. Diabetes Technol Ther. 2021 | 2021 | Japan | Real-time CGM made it possible to track BG trends and prevent dramatic variations in BG, although the rate of insulin infusion changed dynamic. | Non-ICU general wards | Diabetes with Covid |
| Nair, Bala G, Dellinger, E. Patchen, Flum, David R, Rooke, G. Alec, & Hirsch, Irl B. (2020). A Pilot Study of the Feasibility and Accuracy of Inpatient Continuous Glucose Monitoring. Diabetes Care, dc200670. https://doi.org/10.2337/dc20-0670 | 2020 | USA | Pilot Study of ten participants | CGM in Non-ICU general wards | Insulin dependent T1D and T2D |
| Korytkowski M, Antinori-Lent K, Drincic A, Hirsch IB, McDonnell ME, Rushakoff R, Muniyappa R. A Pragmatic Approach to Inpatient Diabetes Management during the COVID-19 Pandemic. J Clin Endocrinol Metab. 2020 Sep 1;105(9):dgaa342. doi: 10.1210/clinem/dgaa342. PMID: 32498085; PMCID: PMC7313952. | 2020 | USA | Case study | Non-ICU general wards | T2D |
| Klonoff DC, Messler JC, Umpierrez GE, Peng L, Booth R, Crowe J, Garrett V, McFarland R, Pasquel FJ. Association Between Achieving Inpatient Glycemic Control and Clinical Outcomes in Hospitalized Patients With COVID-19: A Multicenter, Retrospective Hospital-Based Analysis. Diabetes Care. 2021 Feb;44(2):578-585. doi: 10.2337/dc20-1857. Epub 2020 Dec 15. PMID: 33323475; PMCID: PMC7818335. | 2021 | USA | Retrospective observational study | CGM in non-ICU and ICU | T1D and T2D in Covid Pateints |
| Galindo, Rodolfo J, Migdal, Alexandra L, Davis, Georgia M, Urrutia, Maria A, Albury, Bonnie, Zambrano, Cesar, Vellanki, Priyathama, Pasquel, Francisco J, Fayfman, Maya, Peng, Limin, & Umpierrez, Guillermo E. (2020). Comparison of the FreeStyle Libre Pro Flash Continuous Glucose Monitoring (CGM) System and Point-of-Care Capillary Glucose Testing (POC) in Hospitalized Patients With Type 2 Diabetes (T2D) Treated With Basal-Bolus Insulin Regimen. *Diabetes Care*, dc192073. https://doi.org/10.2337/dc19-2073 | 2020 | USA | Clinical trial | Hospital | T2D |
| Gómez, Ana M, Umpierrez, Guillermo E, Muñoz, Oscar M, Herrera, Felipe, Rubio, Claudia, Aschner, Pablo, & Buendia, Richard. (2016). Continuous Glucose Monitoring Versus Capillary Point-of-Care Testing for Inpatient Glycemic Control in Type 2 Diabetes Patients Hospitalized in the General Ward and Treated With a Basal Bolus Insulin Regimen. *Journal of Diabetes Science and Technology*, *10*(2), 325–329. https://doi.org/10.1177/1932296815602905 | 2016 | USA | No differences in average daily glucose levels were observed between CGM and POC (176.2 ± 33.9 vs 176.6 ± 33.7 mg/dl, P = .828). However, CGM detected a higher number of hypoglycemic episodes than POC | Hospital General Wards | Pateints treated With a Basal Bolus Insulin Regimen |
| Kyi M, Colman PG, Wraight PR, Reid J, Gorelik A, Galligan A, Kumar S, Rowan LM, Marley KA, Nankervis AJ, Russell DM, Fourlanos S. Early Intervention for Diabetes in Medical and Surgical Inpatients Decreases Hyperglycemia and Hospital-Acquired Infections: A Cluster Randomized Trial. Diabetes Care. 2019 May;42(5):832-840. doi: 10.2337/dc18-2342. Epub 2019 Mar 28. PMID: 30923164. | 2019 | Australia | With no use of CGM or FGM early identification and management of inpatients with diabetes decreased hyperglycemia and hospital-acquired infections. | Hospital | T1D and T2D |
| Reutrakul, Sirimon, Genco, Matthew, Salinas, Harley, Sargis, Robert M, Paul, Carlie, Eisenberg, Yuval, Fang, Jiali, Caskey, Rachel N, Henkle, Sarah, Fatoorehchi, Sam, Osta, Amanda, Srivastava, Pavan, Johnson, Alexia, Messmer, Sarah E, Barnes, Michelle, Pratuangtham, Sarida, & Layden, Brian T. (2020). Feasibility of Inpatient Continuous Glucose Monitoring During the COVID-19 Pandemic: Early Experience. *Diabetes Care*, *43*(10), e137–e138. https://doi.org/10.2337/dc20-1503 | 2020 | USA | Pilot study found that CGM use is feasible in noncritically ill COVID-19 patients. Further confirmation is needed through large randomized clinical trials | Hospital for non-critically ill pateints | T1D and T2D |
| Kyi M, Colman PG, Rowan LM, Marley KA, Wraight PR, Fourlanos S. Glucometric benchmarking in an Australian hospital enabled by networked glucose meter technology. Med J Aust. 2019 Aug;211(4):175-180. doi: 10.5694/mja2.50247. Epub 2019 Jun 23. PMID: 31231826. | 2019 | Australia | Glucometric analysis supported by networked glucose meter technology provides detailed inpatient data that could enable local benchmarking for promoting safe diabetes care in Australian hospitals. | Hospital | People with Diabetes in Hospital |
| Kyi M, Wraight PR, Rowan LM, Marley KA, Colman PG, Fourlanos S. Glucose alert system improves health professional responses to adverse glycaemia and reduces the number of hyperglycaemic episodes in non-critical care inpatients. Diabet Med. 2018 Jun;35(6):816-823. doi: 10.1111/dme.13623. Epub 2018 Apr 10. PMID: 29575134. | 2018 | Australia | Use of a novel glucose alert system improved health professional responses to adverse glycaemia and decreased hyperglycaemia in the hospital setting. | Hospital | T1D & T2D in Hospital |
| Fortmann, Addie L, Spierling Bagsic, Samantha R, Talavera, Laura, Garcia, Isabel Maria, Sandoval, Haley, Hottinger, Amiry, & Philis-Tsimikas, Athena. (2020). Glucose as the Fifth Vital Sign: A Randomized Controlled Trial of Continuous Glucose Monitoring in a Non-ICU Hospital Setting. *Diabetes Care*, dc201016. https://doi.org/10.2337/dc20-101 | 2020 | USA | The RT-CGM group demonstrated significantly lower mean glucose (M∆ = -18.5 mg/dL) and percentage of time in hyperglycemia >250 mg/dL (-11.41%) and higher time in range 70-250 mg/dL (+11.26%) compared with UC (P values <0.05). Percentage of time in hypoglycemia was very low. | Non-ICU general wards | T2D |
| Shehav-Zaltzman, G. Segal, G. Konvalina, N. Tirosh, A. Remote Glucose Monitoring of Hospitalized, Quarantined Patients With Diabetes and COVID-19, Diabetes Care Jul 2020, 43 (7) e75-e76; |  |  | CGM for remote real-time diabetes management feasible and added to the quality of care while minimizing health care work exposure risk. | Hospital | T1D and T2D |
| Garg, S. Norman, G. J. Impact of COVID-19 on Health Economics and Technology of Diabetes Care: Use Cases of Real-Time Continuous Glucose Monitoring to Transform Health Care During a Global Pandemic. (2021). Diabetes Technology & Therapeutics, 23(S1), S–15–S–20. https://doi.org/10.1089/dia.2020.0656 | 2021 | USA | Case studies indicate increased infrastructure needs for safe implementation of CGM in the hospital, including staff education, establishing protocols for device placement and replacement, monitoring device accuracy, and integrating CGM readings into the medical record. | Hospital | T1D and T2D |
| Levitt DL, Spanakis EK, Ryan KA, Silver KD. Insulin Pump and Continuous Glucose Monitor Initiation in Hospitalized Patients with Type 2 Diabetes Mellitus. Diabetes Technol Ther. 2018 Jan;20(1):32-38. doi: 10.1089/dia.2017.0250. Epub 2018 Jan 2. PMID: 29293367; PMCID: PMC5770096. | 2018 | USA | Insulin pump and CGM initiation are feasible during hospitalization, although they are labor intensive. Although insulin pump initiation may not lead to improved glycemic control, there is a trend toward CGM detecting a greater number of hypoglycemic episodes. Larger studies are needed to determine whether use of this technology can lower inpatient morbidity and mortality | Non-ICU | T2D |
| Singh LG, Satyarengga M, Marcano I, Scott WH, Pinault LF, Feng Z, Sorkin JD, Umpierrez GE, Spanakis EK. Reducing Inpatient Hypoglycemia in the General Wards Using Real-time Continuous Glucose Monitoring: The Glucose Telemetry System, a Randomized Clinical Trial. Diabetes Care. 2020 Nov;43(11):2736-2743. doi: 10.2337/dc20-0840. Epub 2020 Aug 5. PMID: 32759361; PMCID: PMC7576426. | 2020 | USA | RT-CGM/GTS can decrease hypoglycemia among hospitalized high-risk insulintreated patients with type 2 diabetes | General Wards | T2D |
| Spanakis EK, Levitt DL, Siddiqui T, Singh LG, Pinault L, Sorkin J, Umpierrez GE, Fink JC. The Effect of Continuous Glucose Monitoring in Preventing Inpatient Hypoglycemia in General Wards: The Glucose Telemetry System. J Diabetes Sci Technol. 2018 Jan;12(1):20-25. doi: 10.1177/1932296817748964. Epub 2017 Dec 13. PMID: 29237288; PMCID: PMC5761998. | 2018 | USA | This pilot study indicates that the use of CGM values in hospitalized patients can be successfully transmitted to a monitoring device in the nursing station, improving patient surveillance in insulin treated patients with diabetes. | General Wards | T1D and T2D |
| Ehrhardt N, Hirsch IB. The Impact of COVID-19 on CGM Use in the Hospital. Diabetes Care. 2020 Nov;43(11):2628-2630. doi: 10.2337/dci20-0046. Epub 2020 Sep 25. PMID: 32978180. | 2020 | USA | CGM use may improve glucose control and less PPE utilization for patients with COVID-19; however, the challenge of using CGM in the hospital setting without a dedicated diabetes team or endocrinologist familiar with the technology will be limiting. Furthermore, even for those comfortable with the technology, currently there are no standardized inpatient protocols to address both alerts and predictive alerts. We require evidenced-based protocols on how to best advise nursing staff to respond to glucose and glucose trends | Non-ICU and ICU | T1D and T2D |
| Aleppo G, Webb K. Continuous Glucose Monitoring Integration in Clinical Practice: A Stepped Guide to Data Review and Interpretation. J Diabetes Sci Technol. 2019 Jul;13(4):664-673. doi: 10.1177/1932296818813581. Epub 2018 Nov 19. PMID: 30453772; PMCID: PMC6610596. | 2019 | USA | CGM systems go beyond A1C and fingerstick BGM by bringing glycemic monitoring out of the past, into the present, and looking toward the future. Patients who use CGM have the opportunity to increase their awareness of their own glycemic responses and engagement in the management of their own disease. They can monitor the effects of different foods, times of day, activity levels, and illness on their blood glucose levels. CGM is also beneficial for patients’ caregivers and loved ones who participate in diabetes management. HCPs who incorporate these systems into their clinical practice will gain a valuable tool that provides a comprehensive view of glycemic events, trends, and patterns, enabling them to effectively intervene to improve their patients’ diabetes management and outcomes. | Non-ICU and ICU | T1D and T2D |
| Murray-Bachmann R, Leung TM, Myers AK, Murthi S, Sarbanes M, Ziskovich K, Lesser M, Poretsky L. Reliability of continuous glucose monitoring system in the inpatient setting. J Clin Transl Endocrinol. 2021 Jul 7;25:100262. doi: 10.1016/j.jcte.2021.100262. PMID: 34336598; PMCID: PMC8318984. | 2021 | USA | Prospective cohort study for the non- critical inpatient setting. CGM is reliable and accurate in inpatient setting. | Non-ICU | T1D and T2D |
| Davis GM, Spanakis EK, Migdal AL, Singh LG, Albury B, Urrutia MA, Zamudio-Coronado KW, Scott WH, Doerfler R, Lizama S, Satyarengga M, Munir K, Galindo RJ, Vellanki P, Cardona S, Pasquel FJ, Peng L, Umpierrez GE. Accuracy of Dexcom G6 Continuous Glucose Monitoring in Non-Critically Ill Hospitalized Patients With Diabetes. Diabetes Care. 2021 Jun 7;44(7):1641–6. doi: 10.2337/dc20-2856. Epub ahead of print. PMID: 34099515; PMCID: PMC8323182. | 2021 | USA | Retrospective meta-analysis. The accuracy of Dexcom G6, we compared retrospective matched-pair CGM and capillary point-of-care (POC) glucose data from three inpatient CGM studies (two interventional and one observational) in general medicine and surgery patients with diabetes treated with insulin. | Non-Critically ill patients | T2D |
| Atif Z, Halstrom A, Peragallo-Dittko V, Klek SP. Efficacy of Hybrid Closed-Loop Insulin Delivery System in a Hospital Setting: A Case Series. AACE Clin Case Rep. 2021 Jan 6;7(3):184-188. doi: 10.1016/j.aace.2020.12.013. PMID: 34095484; PMCID: PMC8165117. | 2021 | USA | Case Reports | Non-Critically ill patients | T1D |
| Migdal AL, Spanakis EK, Galindo RJ, Davis G, Singh LG, Satyarengga M, et al. Accuracy and precision of continuous glucose monitoring in hospitalized patients undergoing radiology procedures. J Diabetes Sci Technol. 2020;14:1135–6. | 2020 | USA | Observational study looking at accuracy and precision of Dexcom for patients undergoing radiology procedures | Non-Critically ill patients | T1D and T2D |
| Tripyla A, Herzig D, Joachim D, Nakas CT, Amiet F, Andreou A, et al. Performance of a factory-calibrated, real-time continuous glucose monitoring system during elective abdominal surgery. Diabetes Obes Metab. 2020;22:1678–82. [PubMed] [Google Scholar] | 2020 | Switzerland | No clinically significant adverse events occurred. In conclusion, the Dexcom G6 device showed consistent and acceptable accuracy during elective abdominal surgery, opening new avenues for peri-operative glucose management. | Non-Critically ill patients | Pre-diabetes |
| Nair BG, Dellinger EP, Flum DR, Rooke GA, Hirsch IB. A pilot study of the feasibility and accuracy of inpatient continuous glucose monitoring. Diabetes Care. 2020;43:e168–9. | 2020 | USA | Use of CGM could reduce the number of health care provider contacts and the need for personal protective equipment. | Non-Critically ill patients | adults with diabetes who used intermittent home blood glucose monitoring |
| Gomez AM, Umpierrez GE, Munoz OM, Herrera F, Rubio C, Aschner P, et al. Continuous glucose monitoring versus capillary point-of-care testing for inpatient glycemic control in type 2 diabetes patients hospitalized in the general ward and treated with a basal bolus insulin regimen. J Diabetes Sci Technol. 2015;10:325–9. | 2015 | USA | Use of CGM in type 2 patients hospitalized in the general ward provides accurate estimation of blood sugar levels and is more effective than POC for the detection of hypoglycemic episodes and asymptomatic hypoglycemia. | Non-Critically ill patients | T2D |
| Queirós CS, Alexandre MI, Garrido PM, Correia TE, Filipe PL. Allergic contact dermatitis to IBOA in FreeStyle Libre: Experience from a tertiary care Portuguese hospital. Contact Dermatitis. 2020 Aug;83(2):154-157. doi: 10.1111/cod.13578. Epub 2020 May 19. PMID: 32352577. | 2020 | Portugal | A number of patients experienced skin irritation as a result of the glue holding sensors onto the skin | Non-Critically ill patients | Diabetes (unspecified) |
| Longo RR, Elias H, Khan M, Seley JJ. Use and Accuracy of Inpatient CGM During the COVID-19 Pandemic: An Observational Study of General Medicine and ICU Patients. J Diabetes Sci Technol. 2021 May 10:19322968211008446. doi: 10.1177/19322968211008446. Epub ahead of print. PMID: 33971753. | 2021 | USA | Feasible alternative to POC which reduces staff exposure to COVID risk. Investigation and analysis are necessary for the development of protocols to utilize CGM trend arrows, alerts, and alarms. | ICU and General Wards | Diabetes (unspecified) |
